# Reference-Point Theory: An Account of Individual Differences in Risk Preferences

**DOI:** 10.1177/17456916231190393

**Published:** 2023-09-14

**Authors:** Barbara A. Mellers, Siyuan Yin

**Affiliations:** 1 Department of Psychology, University of Pennsylvania; 2 Department of Marketing, The Wharton School, University of Pennsylvania

**Keywords:** loss aversion, risk aversion, gain seeking, risk seeking, risk preferences, optimism, pessimism, decision-making, emotions, risky choices, individual differences

## Abstract

We propose an account of individual differences in risk preferences called “reference-point theory” for choices between sure things and gambles. Like most descriptive theories of risky choice, preferences depend on two drivers—hedonic sensitivities to change and beliefs about risk. But unlike most theories, these drivers are estimated from judged feelings about choice options and gamble outcomes. Furthermore, the reference point is assumed to be the less risky option (i.e., sure thing). Loss aversion (greater impact of negative change than positive change) and pessimism (belief the worst outcome is likelier) predict risk aversion. Gain seeking (greater impact of positive change than negative change and optimism (belief the best outcome is likelier) predict risk seeking. But other combinations of hedonic sensitivities and beliefs are possible, and they also predict risk preferences. Finally, feelings about the reference point predict hedonic sensitivities. When decision makers feel good about the reference point, they are frequently loss averse. When they feel bad about it, they are often gain seeking. Three studies show that feelings about reference points, feelings about options and feelings about outcomes predict risky choice and help explain why individuals differ in their risk preferences.

Risk is ubiquitous, and preferences for risk are central to the social and behavioral sciences. Since von Neumann and Morgenstern (1944), economists have defined risk preferences using choices between sure things and gambles with equal expected value. Decision makers should prefer the option that maximizes their expected utilities, and their response to risk depends on the shape of their utility function. Risk aversion—a preference for the sure thing—implies a concave downward utility function. Risk taking—a preference for the gamble—implies a concave upward one.

Unlike economists, psychologists focus on why people accept or avoid risk, not what they ought to do. To this end, they have developed descriptive theories, including prospect theory, cumulative prospect theory, security-potential/aspiration theory, and transfer-of-attention theory, to name just a few (Birnbaum, 2008; Kahneman & Tversky, 1979; Lopes, 1987; Lopes & Oden, 1999; Tversky & Kahneman, 1992). In these accounts, the reference point is the status quo and is assumed to be neutral. Decision makers are generally described by a single utility function for outcomes and a single decision-weighting function for probabilities.

We propose a new account of choices between sure things and gambles in which the reference point is the outcome that occurs if risk is rejected (i.e., the sure thing). The sure thing can be charged with positive or negative affect, and these feelings predict hedonic sensitivities (loss aversion and gain seeking). Both hedonic sensitivities and beliefs about risk (optimism and pessimism) drive risk preferences. We now discuss each driver in more detail.

## Hedonic Sensitivity to Change

Loss aversion was proposed by Kahneman and Tversky (1979) to describe the shape of the utility function in prospect theory. Utilities are steeper in the loss domain than the gain domain; losses have greater emotional impact than equivalent gains. This assumption has been used to describe the equity premium puzzle (Benartzi & Thaler, 1995), endowment effects (Kahneman et al., 1991), riskless choices (Tversky & Kahneman, 1991), consumer sensitivity to price changes (Hardie et al., 1993; Putler, 1992; Winer, 1986), New York cabbies’ decisions to stop their shifts (Camerer et al., 1997), and consumer choices among health-care options (Samuelson & Zeckhauser, 1988).

It is therefore unusual that when loss aversion was tested using ratings of pleasure and pain, the results were decidedly mixed. In a typical study, people judge how good (bad) they imagine feeling if they won (lost) a monetary amount, and the absolute magnitudes of ratings are compared. Some studies have shown that the impact of a loss is greater than that of a gain (Baumeister et al., 2001; Rozin & Royzman, 2001). Other stuides have found that the impacts of losses and gains are similar (Mellers et al., 1997). Still others have demonstrated that the intensity of a gain exceeds that of a loss (Harinck et al., 2007; Kermer et al., 2006; Mellers & Ritov, 2010; Peters et al., 2003).

Mellers et al. (2021) suggested these inconsistencies could happen if the reference point is not affectively neutral. If so, tests of loss aversion require one to obtain judged feelings of the reference point, the positive outcome, and the negative outcome. Only then can one compare the hedonic impact of a positive change to that of a negative one.

To illustrate, suppose a consumer expects to pay $100 for a product. That consumer discovers a better price (e.g., $80) in one store and a comparable worse price (e.g., $120) in another. To find out whether the consumer is loss averse, one needs to measure the consumer’s feelings about the expected price, F($100); the better price, F($80); and the worse price, F($120). If the absolute value of the negative change, |F($120) – F($100)|, exceeds that of the positive change, |F($80) – F($100)|, the customer is loss averse. If the opposite occurs, the customer is gain seeking.

Mellers et al. (2021) manipulated the valence of the reference point, measured judged pleasure and discovered two systematic patterns. Reference points were expected exam grades of B or D, expected exercise goals of seven or 27 sit-ups, and expected headphone prices of $120 or $310. Participants were asked how they would feel about the expected outcome, a better outcome, and an equal-distant worse outcome using a scale from *extremely bad* (–5) to *extremely good* (5).

For example, one group was asked,

Imagine you are a student taking a challenging course. You just took the final exam and expect a B. You get it. How would you feel? Suppose that, instead of a B, you receive an A. How would you feel? Now suppose that, instead of a B, you get a C. How would you feel?

Another group was asked,

Imagine you are a student taking a challenging course. You just took the final exam and you expect a D. You get it. How would you feel? Suppose that, instead of a D, you receive a C. How would you feel? Now suppose that, instead of a D, you get an F. How would you feel?

**Fig. 1.**
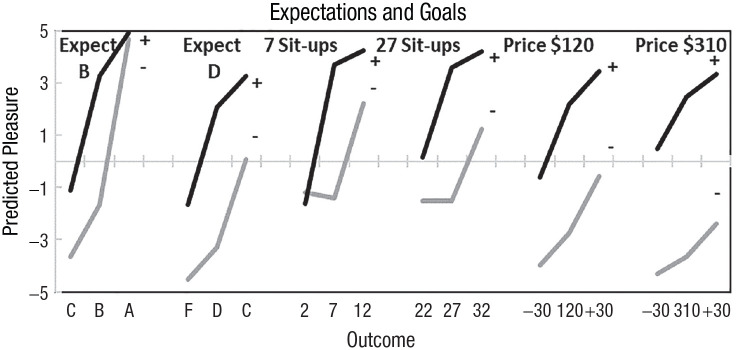
Judged pleasure when reference points are expected grades, exercise goals, and expected headphone prices. Black lines display loss aversion among participants who felt positively about the reference point (+). Gray lines show gain seeking among participants who felt negatively about the reference point (−). From Mellers et al. (2021).

Participants differed greatly in their emotional reactions to the reference point. Some felt that getting a B was good, while others thought it was extremely bad. Mellers et al. (2021) sorted participants according to the sign of their feelings about the reference point. Judged pleasure is shown in Figure 1 against the worst, the expected, and the best outcome. For participants who felt good about the expected outcome (center points in each black line), negative changes had greater hedonic impact than positive changes, consistent with loss aversion. For participants who felt bad about the expected outcome (center points in each gray line), positive changes have greater hedonic impact than negative changes, consistent with gain seeking. Both loss aversion and gain seeking occur, depending on the valence of the reference point. In this article, we examine whether these patterns in hedonic sensitivities also occur in risky choice.

## Beliefs About Risk

Both psychologists and economists have investigated beliefs in risky choice using functions that transform objective probabilities into decision weights (Luce & Fishburn, 1991; Quiggin, 1982; Schmeidler, 1989; Yaari, 1987). The decision-weighting function in cumulative prospect theory is inverse S-shaped, first concave then convex. Kahneman and Tversky (1979) and many others have argued that smaller (larger) probabilities are weighted more (less) heavily (Prelec, 1998; Tversky & Kahneman, 1992; Wu & Gonzalez, 1996).

A key feature in many decision-weighting functions is rank dependency; the weight of an outcome depends not only on the probability of its occurrence but also on its rank order in the set of possible outcomes. E. U. Weber and Kirsner (1997) summarized three reasons that weights might be rank dependent. First, rank dependency could stem from perceptual processing if decision makers attend to more extreme outcomes (Tversky & Kahneman, 1992). Second, rank-dependency could occur if decision makers wish to minimize an asymmetric loss function (Birnbaum et al., 1992). Third, rank-dependency could arise if decision makers have different but stable trait dispositions. Lopes (1984, 1987) argued that people who focus on the worst outcome are security-minded, and those who focus on the best outcome are potential-minded. We build on Lopes’s view of rank dependency but without the assumption that beliefs are stable traits. Beliefs reflect optimism or pessimism about a particular choice. Any further claims would require additional tests beyond the scope of this paper.

## Reference-Point Theory

Consider a choice between $500 for sure and a gamble with 50/50 chances of $250 and $750. We ask decisioned makers how they would feel about accepting each choice option and receiving each outcome in the gamble on a rating scale from *extremely good* to *extremely bad*. Feelings about the sure thing and the gamble are F($500) and F(G), respectively, and feelings about gamble outcomes are F($250) and F($750). Then we asked for a choice.

Reference-point theory makes six assumptions. First, the rank order of judged feelings about options predicts choice. If F($500) > F(G), the decision maker is risk averse. If F(G) > F($500), the decision maker is risk seeking. If F($500) = F(G), the decision maker is indifferent between the options.

Second, the reference point is the judged feeling about the sure thing, F($500), a natural anchor against which decision makers evaluate the gamble.

Third, hedonic sensitivities are estimated from judged feelings. If |F($250) – F($500)| > |F($750) – F($500)|, the decision maker is loss averse. If the opposite occurs, the decision maker is gain seeking.

Fourth, feelings about the gamble are a weighted average of outcome feelings:

(1) F(G)=w×F($250)+(1−w)×F($750),

where *w* is the decision weight of the worst outcome.

Fifth, we estimate the decision weight of the worst outcome by solving for *w*:

(2) w=[F(G)–F($750)]/[F($250)–F($750)].

If the objective probability of the worst outcome is .5 and the decision weight is greater than .5, the decision maker is pessimistic about risk. If the decision weight is less than .5, the decision maker is optimistic about risk because the gamble is binary and weights sum to 1.0.

Equation 2 has no degrees of freedom, which means it is always possible to estimate a decision weight. However, because decision weights must fall between 0 and 1 inclusive, this assumption is testable. Whenever weights fall outside that range, reference-point theory cannot account for the data.^1^

The sixth assumption is that individual differences in hedonic sensitivities and beliefs about risk provide the reasons for risk preferences. These drivers are conceptually independent (see the Appendix in the Supplemental Material available online), so all combinations are possible. However, reference-point theory predicts that only some of the combinations are consistent with risk-averse and risk-seeking preferences.

## Capturing Individual Differences

To examine individual differences, we consider combinations of hedonic sensitivities and beliefs about risk as shown in Table 1. Rows are beliefs, and columns are hedonic sensitivities. Cell entries are predicted risk preferences from reference-point theory (see the Appendix in the Supplemental Material). Table 1 simplifies all possible outcomes because decision makers can also have equal hedonic sensitivities and/or calibrated decision weights (equal to objective probabilities), and if so, we say that driver is symmetric (neutral) and favors neither risk aversion nor risk seeking.

**Table 1.** Pairs of Reasons and Predicted Risk Preferences

	LA	GS
PES	RA	RA or RS
OPT	RA or RS	RS

Note: RA and RS are predicted risk preferences according to reference-point theory. LA = loss aversion; GS = gain seeking; PES = pessimism about risk; OPT = optimism about risk; RA = risk averse; RS = risk seeking.

Table 1 shows that when decision makers have two consistent reasons for a risk preference, reference-point theory makes a single prediction. That is, decision makers who are loss averse and pessimistic can be risk averse, but not risk seeking. Decision makers who are gain seeking and optimistic can be risk seeking, but not risk averse. Decision makers can also have inconsistent reasons for their risk preferences, as shown in the off-diagonal in Table 1. Reference-point theory predicts that these decision makers can be risk-averse or risk-seeking. Finally, decision makers can have one reason for a risk preference with the other being neutral (not shown in Table 1). In this case, the theory predicts that risk preferences are consistent with the single reason (i.e., pessimists with equal hedonic contrasts or loss averters with calibrated beliefs should be risk averse).

Table 1 shows that decision makers can be risk averse for three pairs of reasons. They can be (a) “pessimistic loss averters” (PLAs) with two consistent reasons for risk aversion. They believe the worst outcome is likelier, and they anticipate greater pain from a negative change than pleasure from a comparable positive change. It is worth noting that decision makers with one reason for risk aversion (just loss aversion or just pessimism) are also predicted to be risk averse. (b) “Pessimistic gain seekers” (PGSs) have one reason for risk aversion (pessimism) and one for risk seeking (gain seeking). Reasons conflict; pessimism motivates risk aversion, and gain seeking motivates risk seeking. These decision makers believe the worse outcome is likelier to occur, but they also anticipate greater pleasure from a positive change than pain from a comparable negative one. If pessimism is more important, they are likely to be risk averse. If gain seeking is more important, they are likely to be risk seeking. Finally, (c) “optimistic loss averters” (OLAs) have one reason for risk aversion (loss aversion) and one reason for risk seeking (optimism). Again, reasons conflict; optimism encourages risk seeking, and loss aversion promotes risk aversion. These decision makers believe the best outcome is likelier to occur, but they anticipate more intense pain from a negative change than pleasure from a positive one. If optimism is more important, they are risk seeking. If loss aversion is more important, they are risk averse.

Table 1 further shows three pairs of reasons for risk seeking. Decision makers can be (a) PGSs with one reason for risk seeking (gain seeking) and one reason for risk aversion (pessimism), (b) OLAs with one reason for risk seeking (optimism) and one reason for risk aversion (loss aversion), or (c) “optimistic gain seekers” (OGSs) who have mutually reinforcing reasons for risk seeking. They believe that they are lucky (i.e., the best outcome is likelier than the worst one), and they anticipate more intense pleasure from a positive change than pain from a negative one.

We suspect decision makers with two consistent reasons have stronger risk preferences and are less conflicted with their choices than decision makers with one reason (i.e., just pessimism or just loss aversion for risk averters). Decision makers with one reason may also have stronger preferences than decision makers with inconsistent reasons. That is, reasons for risk preferences may predict the strength of decision makers’ preferences. We speculate even further and suggest that risk preferences fall along a continuum that ranges from risk averters with two consistent reasons, risk averters with one reason, risk averters with conflicting reasons, risk seekers with conflicting reasons, risk seekers with one reason, and finally, risk seekers with two consistent reasons.

## Overview

In this article, we build on the following ideas: (a) When people choose between sure things and binary gambles, the reference point is the outcome that occurs if risk is rejected; (b) risk preferences depend on hedonic sensitivities and beliefs about risk; and (c) hedonic sensitivities are predictable from the valence of the reference point.

To assess hedonic sensitivity, we designed gambles with outcomes that are equidistant from the sure thing but opposite in sign. We elicit choices and measure the judged pleasure of the sure thing, the gamble, the best gamble outcome and the worst gamble outcome. We expect to find patterns similar to those in Figure 1 when we examine effects of positive and negative changes around the sure thing.

In three studies, we examine whether reference-point theory can describe individual differences in risk preferences. In all studies, we manipulate or measure the valence of the reference point, investigate effects of hedonic sensitivities on risk preferences, and explore individual differences in reasons for risk preferences. In Study 1, we experimentally manipulate reference points and examine hedonic sensitivities, beliefs, and risk preferences. Participants make choices between a sure job and a risky job. Jobs vary in salaries, commute times, average winter temperatures, or city-safety indices.

In Study 2, we manipulate reference points using framing effects from Tversky and Kahneman’s (1981) famous disease problem.[AQ10] According to prospect theory, reference points are 0 lives saved and 0 lives lost. Participants should be risk averse in the lives-saved frame and risk seeking in the lives-lost frame. In reference-point theory, reference points are 200 lives saved and 400 lives lost. We expect most people will judge 200 lives saved positively and 400 lives lost negatively. Participants can be either risk averse or risk seeking in each frame, although preferences are likelier to be risk averse if decision makers feel positively about the reference point and risk seeking if decision makers feel negatively about the reference point.

In Study 3, we explore preferences for hypothetical fair 50/50 gambles with stakes ranging from $5 to $500. Decision makers can accept or reject the gamble. The reference point is gamble rejection. We measure participants’ choices and feelings about accepting the gamble, rejecting the gamble and winning or losing gamble stakes. Prospect theory predicts decision makers should avoid fair 50/50 gambles. Reference-point theory predicts that if gamble rejection is judged positively, decision makers are likelier to be both loss averse and risk averse. But if gamble rejection is judged negatively, decision makers are likelier to be gain seeking and risk seeking.

## Study 1: Manipulating Reference Points for Jobs That Vary in Life Dimensions

Participants were presented with choices between a sure job (the reference point) and a risky job with outcomes that were equidistant from the sure thing but opposite in sign. Stimuli were adapted for choices from judgment tasks in Mellers et al. (2021). Participants were randomly assigned to one of eight conditions based on two reference points (Better and Worse) × 4 dimensions (Salary, Commute Time, Average Winter Temperature, and City-Safety Index). Decision makers made one choice and four ratings of pleasure—two for each option and two for each outcome of the risky option.

### Method

This study was preregistered (https://aspredicted.org/V96_JHX) for 800 participants.

#### Stimuli and measures

Table 2 shows manipuated reference points—better and worse—along four life dimensions. To illustrate, suppose participants are given the better reference point for average winter temperature. They are asked, “Imagine you have a choice between a job with an average winter temperature of 40F or a job with 50/50 chances that the average winter temperature is 20F or 60F. Which job would you prefer?” Then we asked, “How would you feel if you accepted the job with 40F average winter temperature? The risky job?” Finally, they rated their feelings about gamble outcomes when we asked, “How would you feel if you took the risky job and the average winter temperature was 60F? How would you feel if you took the risky job and the average winter temperature was 20F?” Ratings were made on a bipolar scale from *extremely bad* (–5) to *extremely good* (5).

**Table 2.** Better and Worse Reference Points for Jobs Varying in Four Life Dimensions

Reference point	Salary	Commute	Average winter temperature	City-safety index
Better	$50,000	20 min	40° F	85%
Worse	$30,000	50 min	0° F	65%

Note: Positive and negative changes in risky job are ±$10,000, 20 min, 20° F, and 10%. The city-safety index is the percentage of U.S. cities that are less safe, so higher numbers are better.

#### Participants and payments

We excluded participants using the same preregistered criteria across all studies. Exclusion occurred if participants (a) failed the attention check, (b) did not complete the survey, or (c) gave the same responses to the worst outcome, the reference point, and the best outcome (e.g., jobs with an average winter temperature of 20° F, 40° F, and 60° F). Exclusions were 3% of the sample, which left us with 776 participants. Our remaining sample was 49% female and had an average age of 40 (range = 18–80). They were paid $0.50 for approximately 3 min of work.

### Results

#### Valence of the reference point

**Fig. 2:**
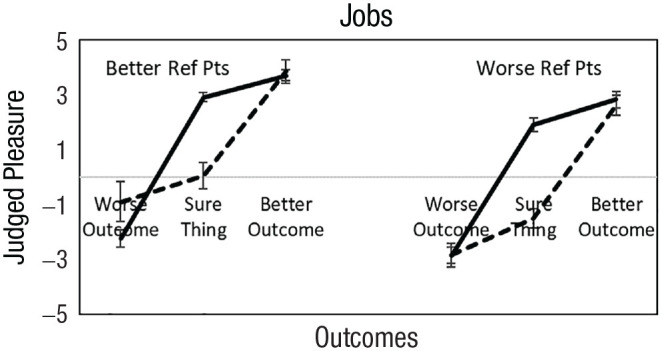
Judged pleasure shown against the reference point, a negative change, and positive change for better and worse reference-point conditions. Solid lines are loss averters, and dashed lines are gain seekers. Participants with equal hedonic contrasts (11%) are not shown. Error bars are 95% confidence intervals around means. Results are pooled across life dimensions.

We begin by examining patterns of loss aversion and gain seeking relative to feelings about the sure thing. Figure 2 shows judged feelings associated with a worse outcome, the sure thing, and a better outcome when reference points were better and worse. Participants with equal hedonic contrasts (11%) are not shown. We found loss aversion and gain seeking among both risk averters and risk seekers in both frames. Next, we examine whether the valence of the reference point predicts hedonic sensitivities and risk preferences.

We sorted participants according to the valence of their judged feelings about the reference point. With better reference points, 81% of participants judged the certain job as positive. The rest judged it as neutral (13%) or negative (6%). With worse reference points, 52% of participants judged the sure job as positive, and the rest felt neutral (16%) or negative (32%). Although the majority felt good about the reference point in both conditions (better and worse), relatively more judged it positively when reference points were better than worse (81% vs. 52%), *t*(2,311) = 38.17, *p* < .001.

Table 3 shows hedonic sensitivities and risk preferences for participants who judged the reference point as positive, neutral, and negative for better and worse conditions and the averages over reference point conditions. Consistent with our hypotheses, participants who rated the certain job positively (vs. negatively) were more often loss averse (84% vs. 13%), *t*(704) = 15.22, *p* < .001, and risk averse (80% vs. 56%), *t*(704) = 4.72, *p* < .001. Participants who rated the sure job negatively (vs. positively) were more often gain seeking (77% vs. 9%), *t*(704) = 13.72, *p* < .001, and risk seeking (44% vs. 20%), *t*(704) = 4.72, *p* < .001. Percentages of hedonic sensitivities and risk preferences for participants with neutral feelings fell between participants who felt positively and negatively.

**Table 3.** Hedonic Sensitivities and Risk Preferences

	LA	RA	GS	RS
Positive RPs (*N* = 517)				
Better RPs (*n* = 315)	83%	82%	8%	18%
Worse RPs (*n* = 202)	85%	78%	10%	22%
Average	84%	80%	9%	20%
Neutral RPs (*N* = 112)				
Better RPs (*n* = 50)	34%	72%	34%	28%
Worse RPs (*n* = 62)	48%	71%	29%	29%
Average	42%	71%	31%	29%
Negative RPs (*N* = 146)				
Better (*n* = 23)	9%	48%	100%	52%
Worse (*n* = 123)	14%	58%	73%	42%
Average	13%	56%	77%	44%

Note: “Positive,” “Neutral,” and “Negative” refer to judged feelings about the RPs. “Better” and “Worse” refer to manipulated RPs across life dimensions (see Table 2). Averages are weighted based on numbers of participants in each condition. RPs = reference points; LA = loss averse; RA = risk averse; GS = gain seeking; and RS = risk seeking.

We also performed the analyses in Table 3 separately for each life dimension. Loss aversion and risk aversion were likelier to occur among participants who judged reference points positively (vs. negatively) on all dimensions. Gain seeking and risk seeking tended to be more likely when participants judged reference points negatively (vs positively). (See the Appendix in the Supplemental Material).

#### Individual differences

What were the reasons for the preferences of risk averters and risk seekers? Table 4 shows pairs of reasons as pessimistic loss averters (PLA), pessimistic gain seekers (PGS), optimistic loss averters (OLA) and optimistic loss averters (OGS). We focus on averages over better and worse conditions.^2^

**Table 4.** Pairs of Reasons for Risk Preferences for Participants With Positive, Neutral, and Negative Feelings About the Reference Point

	Risk averters				Risk seekers			
Positive RPs (*N* = 349)	PLA	PGS	OLA	OGS	PLA	PGS	OLA	OGS
Better (*n* = 305)	67%	3%	11%	0%	2%	2%	8%	7%
Worse (*n* = 44)	0%	9%	0%	0%	25%	7%	30%	30%
Average	59%	3%	10%	0%	5%	3%	11%	10%
Neutral RPs (*N* = 216)								
Better (*n* = 19)	5%	0%	95%	0%	0%	0%	0%	0%
Worse (*n* = 197)	71%	6%	7%	1%	1%	5%	2%	8%
Average	65%	5%	15%	1%	1%	5%	1%	7%
Negative RPs (*N* = 178)								
Better (*n* = 58)	48%	12%	14%	3%	0%	3%	0%	19%
Worse (*n* = 120)	18%	32%	3%	5%	0%	12%	0%	32%
Average	28%	25%	6%	4%	0%	9%	0%	28%

Note: “Positive,” “Neutral,” and “Negative” refer to judged feelings about the RPs. “Better” and “Worse” are RP conditions (see Table 2). Averages are weighted based on the numbers of participants in each condition. RPs = reference points; PLA = Pessimistic Loss Averters; PGS = Pessimistic Gain Seekers; OLA = Optimistic Loss Averters; OGS = Optimistic Gain Seekers.

First, reference-point theory says that risk averters cannot be OGSs, and risk seekers cannot be PLAs. Relatively few risk averters were OGSs; 0%, 1%, and 4% were OGSs when they felt positively, neutrally, and negatively about the reference point, respectively. Moreover, relatively few risk seekers were PLAs; 5%, 1%, and 0% were PLAs when risk seekers felt positively, neutrally, and negatively about the sure thing.

Next, we examine the more common reasons for preferences. When decision makers felt positively about the sure thing, risk averters were PLAs (59%), and risk seekers were OLAs (11%). Loss aversion was common across both groups. When decision makers felt negatively about the sure thing, risk averters tended to be PLAs (28%) and PGSs (25%), and risk seekers were OGSs (28%). Gain seeking was common across both groups. In general, hedonic sensitivities were associated with feelings about the reference point—decision makers were often loss averse when they felt good about the reference point and gain seeking when they felt bad about it. In addition, risk averters were frequently pessimists, and risk seekers optimists.

#### Predicting risk preferences

To find out how well reference-point theory accounted for the data, we estimated the weight of the worst outcome for each participant using Equation 2 and found that 5% of participants had weights outside 0 to 1. Next, we compared the rank order of judged feelings to risk preferences. We discovered that 5% of participants made choices that were inconsistent with their judged feelings about options. Another 10% had judged feelings that were identical, so we assumed reference-point theory was correct for 5% and incorrect for 5%. Thus, 15% of participants (5% + 5% + 5%) were inconsistent with reference-point theory, or 85% were successfully predicted. Of those participants, 71% were risk averters, and 14% were risk seekers.

We compared our account to that of prospect theory. Prospect theory presumably predicts risk aversion because, if reference points are zero, values of all life dimensions (with one excemption) are in the gain domain.^3^ Prospect theory predicted 74% of participants (risk averters) accurately, significantly less than the percentage predicted by reference-point theory, *t*(774) = 4.70, *p* < .001. Reference-point theory also provides reasons for preferences. Hedonic sensitivities were largely determined by decision makers’ feelings about the reference point and were similar across risk preferences. Beliefs about risk tended to vary with risk preferences.

## Study 2: Using Frames to Manipulate Reference Points

Framing effects occur when mathematically equivalent problems with different outcome descriptions lead to systematic reversals of choice (Tversky & Kahneman, 1981). We use the classic disease problem proposed by Tversky and Kahneman (1981) to test reference-point theory. Much is known about effect sizes for these choices. Effect size depends on the desirability of the victims (Levin & Chapman, 1990), the presentation format (Fagley & Miller, 1997), the temporal proximity of outcomes (McElroy & Mascari, 2007), whether participants provide rationales (LeBoeuf & Shafir, 2003; Miller & Fagley, 1991; Sieck & Yates, 1997; Takemura, 1994), and individual-difference variables, such as need for cognition (Chatterjee et al., 2000; Curseu, 2006; Simon et al., 2004; Smith & Levin, 1996; Zhang & Buda, 1999), competence (Bruine de Bruin et al., 2007), and style of thought processes (McElroy & Seta, 2003).

In the disease problem, participants in both frames are told, “Imagine that the U.S. is preparing for the outbreak of an unusual Asian disease which is expected to kill 600 people. Two alternative programs to combat the disease have been proposed.” Then they are randomly assigned to a frame.

In the lives-saved frame, participants read,

Assume that the exact scientific estimate of the consequences of the programs are as follows:

If Program A is adopted, 200 people will be saved.

If Program B is adopted, there is a 1/3 probability that 600 people will be saved and a 2/3 probability that no people will be saved.

Which of the two programs would you prefer?

In the lives-lost frame, participants read,

Assume that the exact scientific estimate of the consequences of the programs are as follows:

If Program C is adopted, 400 people will die. If Program D is adopted, there is a 1/3 probability that nobody will die and a 2/3 probability that 600 people will die.

Which of the two programs would you prefer?

Tversky and Kahneman (1981) stated, “In prospect theory, outcomes are evaluated as deviations (gains and losses) from a neutral reference point which is assigned a value of zero” (p. 454). The assumed reference points are 0 lives saved and 0 lives lost, both of which are value neutral. In reference-point theory, the reference points are sure things—200 lives saved and 400 lives lost—and these outcomes can be affectively charged. If decision makers rate 200 lives saved positively and 400 lives lost negatively, framing effects will reverse the valence of the reference point.

To assess hedonic sensitivities, we need outcomes equidistant from the sure thing but opposite in sign. But in the disease problem, gamble outcomes differ in their distance from the sure thing. So, we included an additional outcome in each frame of 400 lives saved and 200 lives lost. Finally, gamble probabilities are .67/.33, not .50/.50, which implies that reference-point theory places no constraints on reasons for risk preferences (see Appendix in the Supplemental Material). Despite the lack of predictions, we can still examine which reasons drive risk preferences.

### Method

This study was preregistered (https://aspredicted.org/VMZ_H4Z) for 700 workers. Prolific workers were randomly assigned to one of two between-subjects frames (lives saved or lives lost). The procedure was similar that of Study 1.

#### Stimuli and measures

Participants were given a single frame and made a single choice. They were asked, “How would you feel about accepting the sure (risky) option?” In the lives-saved frame, participants were also asked, “How would you feel if 0 lives were saved? 600 lives were saved? Although 400 lives saved was not an outcome, how would you feel if it occurred?” In the lives-lost frame, we asked, “How would you feel if 600 lives were lost? 0 lives were lost? Although 200 lives lost was not an outcome, how would you feel if it occurred?” Responses were made on a scale from *extremely bad* (–5) to *extremely good* (5).

#### Participants and payments

We excluded participants who (a) failed the attention check, (b) did not complete the survey, or (c) gave the same responses to the worst outcome, the reference point, and the best outcome. Ten percent of participants were excluded, leaving us with 630 participants. The sample was 49% females and had an average age of 40 (range = 18–80). Participants were paid $0.50 for approximately 3 min of work.

### Results

Results were consistent with the predictions of Tversky and Kahneman (1981). The majority of participants were risk averse in the lives-saved frame (82%) and risk seeking in the lives-lost frame (63%).

#### Valence of the reference point

**Fig. 3.**
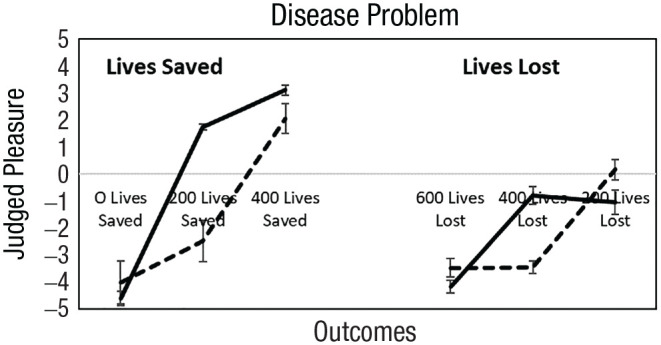
Judged feelings about 0, 200, and 400 lives saved (left) and 600, 400, and 200 lives lost (right). Reference points are the sure things (200 lives saved and 400 lives lost). Participants with equal hedonic contrasts (10%) are not shown. Loss averters are solid lines, and gain seekers are dashed lines. Error bars are 95% confidence intervals.

Once again, we investigated whether participants tended to be loss averse or gain seeking relative to the sure thing. Figure 3 presents judged feelings about 0, 200, and 400 lives saved and 600, 400, and 200 lives lost. Participants with equal hedonic contrasts (10%) are not shown. We found the same patterns (Fig. 3) as we did in Study 1 (Fig. 2) when reference points were manipulated.

Neither 0 lives saved nor 0 lives lost is affectively neutral, as assumed by Tversky and Kahneman (1981). Zero lives saved was extremely bad (–4.52 on a response scale of –5 to 5), and 0 lives lost was extremely good (4.62 on the response scale and not show in Figure 3). Next, we tested hypotheses about reference-point valence, hedonic sensitivities, and risk preferences.

We categorized participants according to their feelings about the reference point. In the lives-saved frame, 70%, 11%, and 19% of participants judged 200 lives saved as positive, neutral, and negative, respectively. In the lives-lost frame, 11%, 10%, and 79% of participants judged 400 lives lost as positive, neutral, and negative, respectively.

Table 5 shows the reference point results. When feelings about the reference point were positive, participants were more likely to be loss averse (98% vs. 29%), *t*(560) = 16.56, *p* < .001, and risk averse (80% vs. 41%), *t*(560) = 9.50, *p* < .001. When feelings about the reference point were negative, participants were more likely to be gain seeking (55% vs. 0%), *t*(560) = 13.98, *p* < .001, and risk seeking (59% vs. 16%), *t*(560) = 9.50, *p* < .001. When participants felt neutral about the reference point, hedonic sensitivities and risk preferences fell between those in positive and negative categories, as expected.

**Table 5.** Hedonic Sensitivities and Risk Preferences

	LA	RA	GS	RS
Positive RPs (*N* = 256)				
Lives saved (*n* = 222)	98%	83%	1%	17%
Lives lost (*n* = 34)	100%	59%	0%	41%
Average	98%	80%	0%	16%
Neutral RPs (*N* = 66)				
Lives saved (*n* = 34)	71%	71%	6%	29%
Lives lost (*n* = 32)	56%	38%	22%	62%
Average	64%	55%	14%	45%
Negative RPs (*N* = 308)				
Lives saved (*n* = 62)	33%	71%	46%	29%
Lives lost (*n* = 246)	28%	34%	58%	66%
Average	29%	41%	55%	59%

Note: “Positive,” “Neutral,” and “Negative” refer to judged feelings about the RPs. Averages are weighted based on numbers of participants in frames. RPs = reference points; LA = loss averse; RA = risk averse; GS = gain seeking; RS = risk seeking.

#### Individual differences

Table 6 shows pairs of reasons for risk averters and risk seekers separately for reference-point valence and averaged over frames. When the reference point was judged positively, risk averters were more likely to be loss averters (PLAs and OLAs), and risk seekers were loss averse (OLAs). When the reference point was judged negatively, risk averters were both loss averse and gain seeking (PLAs and PGSs), and risk seekers were gain seeking (PGS and OGS). Overall trends were loss aversion when reference points were positive, and gain seeking when reference points were negative. Beliefs about risk were more complicated. When the reference point was judged positively, risk averters were optimists and pessmists, and risk seekers were optimists. When the reference point was judged negatively, risk averters were pessimists and risk seekers were optimists and pessimists.

**Table 6.** Reasons for Risk Preferences With Positive, Neutral, and Negative Feelings About Reference Points

	Risk averters				Risk seekers			
Positive RPs (*N* = 256)	PLA	PGS	OLA	OGS	PLA	PGS	OLA	OGS
Lives saved (*n* = 222)	35%	2%	45%	1%	0%	0%	10%	1%
Lives lost (*n* = 34)	21%	6%	12%	18%	0%	3%	6%	18%
Average	33%	2%	41%	3%	0%	0%	10%	4%
Neutral RPs (*N* = 61)								
Lives saved (*n* = 32)	30%	3%	24%	0%	0%	0%	27%	0%
Lives lost (*n* = 29)	7%	17%	3%	3%	7%	14%	14%	17%
Average	19%	10%	15%	2%	3%	6%	21%	8%
Negative RPs (*N* = 304)								
Lives saved (*n* = 59)	44%	5%	10%	2%	8%	0%	15%	2%
Lives lost (*n* = 245)	6%	17%	1%	1%	5%	21%	7%	22%
Average	14%	14%	3%	1%	6%	17%	8%	18%

Note: “Positive,” “Neutral,” and “Negative” refer to judged feelings about the RPs. Averages are weighted based on numbers of participants in frames. RP = reference point; PLA = pessimistic loss averter; PGS = pessimistic gain seeker; OLA = optimistic loss averter; OGS = optimistic gain seeker.

Underlying reasons for choices in the disease problem were somewhat similar to those in Study 1. Hedonic sensitivities were associated with feelings about reference points. When reference points were judged positively, many participants were loss averse, and when reference points were judged negatively, many participants were gain seeking. However, unlike Study 1, many risk averters and risk seekers were both optimists and pessimists.

#### Predicting risk preferences

We fit reference-point theory to the data and found that 6% of participants had weights outside the range of 0 to 1. An additional 4% had choices that were inconsistent with judged feelings about options, and 14% said their feelings about options were the same. We assumed half were consistent with reference-point theory and that half were not. A total of 17% (6% + 4% + 7%) of participants could not be accounted for by reference-point theory, and 83% were accurately predicted. In prospect theory, decision makers should be risk averse in the lives-saved frame and risk seeking in the lives-lost frame. Prospect theory accurately accounted for 73% of participants across frames. A comparison of fits shows that reference-point theory predicted more the choices of more participants than prospect theory (73% vs. 83%), *t*(628) = 6.05, *p* < .001. Now we turn to a study in which the valence of the reference point was measured rather than manipulated.

## Study 3: Measuring Reference Points With Fair 50/50 Gambles

In Study 3, we used fair 50/50 gambles with varying stakes in a between-subjects design. Participants could accept or reject a gamble. According to prospect theory, the reference point is the status quo, or $0. In our account, the reference point is rejection of the gamble.^4^ Refusal of risk is not the same as the status quo. Participants who judged gamble rejection positively are likelier to be loss averse (greater pain from negative change) and risk averse (choose the sure thing). Participants who judged the gamble rejection negatively are likelier to be gain seeking (greater pleasure from positive change) and risk seeking (choose the gamble).

### Method

We recruited 1,352 workers on Prolific Academic, who were randomly assigned to one of six conditions with stakes of $5, $10, $25, $50, $100, or $500.

#### Stimuli and measures

Participants were told, “Imagine you were offered a chance to play a gamble with a 50% chance of winning $*X* and a 50% chance of losing $X. Would you take it?” *X* ranged from $5 to $500. Then we asked, “How would you feel if you took the gamble? How would you feel if you didn’t take it?” Finally, we asked, “How would you feel if you won $*X*? Lost $*X*?” Responses were made on a bipolar scale from –5 (*extremely bad*) to 5 (*extremely good*).

#### Participants and payments

We did not preregister this study, but we used the same exclusion criteria as in Studies 1 and 2. Of the 1,352 workers, 152 were excluded (11%), leaving us with 1,200 participants. The sample was 52% female and ranged in age from 18 to 77, with an average of 32. All were paid $0.35 for approximately 2 min of work.

### Results

#### The valence of the reference point

**Fig. 4.**
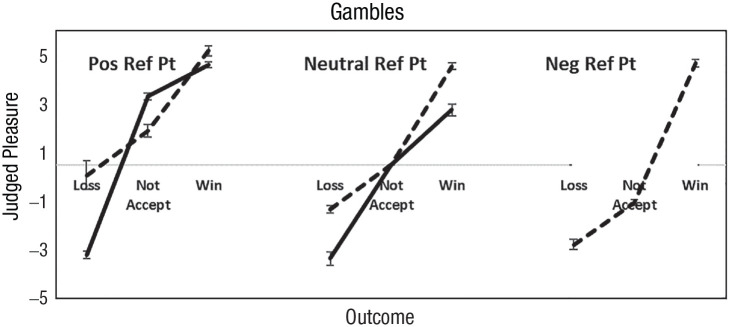
Judged feelings about losing, not accepting the gamble, and winning when gamble rejection was judged positively, neutral, and negatively. Loss averters are solid lines, and gain seekers are dashed lines. No participants who felt bad about rejecting the gamble were loss averse. Error bars are 95% confidence intervals.

We sorted participants according to the sign of their feelings about the reference point and their hedonic sensitivities. There were 40%, 42%, and 18% of participants who felt positively, neutral and negatively about rejecting the gamble, respectively. Ten percent of participants (not shown) had equal hedonic contrasts. Figure 4 shows hedonic sensitivities. There were no loss averters who felt negatively about gamble rejection. The same patterns in Figures 1 to 3 appear with fair 50/50 gambles when feelings about gamble rejection is the reference point.

Table 7 presents hedonic sensitivities and risk preferences for the three categories of valence about the reference point. When participants felt positively (vs negatively) about rejecting the gamble, they were likelier to be loss averse (91% vs. 0%), *t*(976) = 57.5, *p* < .001, and risk averse (80% vs. 36%), *t*(976) = 10.04, *p* < .001. When they felt negatively (vs positively) about rejecting the gamble, they were likelier to be gain seeking (99% vs. 5%), *t*(976) = 58.78, *p* < .001, and risk seeking (64% vs. 20%), *t*(976) = 27.85, *p* < .001. Hedonic sensitivities and risk preferences were predictable from the valence of the reference point.

**Table 7.** Hedonic Sensitivities and Risk Preferences

	LA	RA	GS	RS
Positive RPs (*N* = 492)	91%	80%	5%	20%
Neutral RPs (*N* = 222)	14%	47%	49%	53%
Negative RPs (*N* = 486)	0%	36%	99%	64%

Note: “Positive,” “neutral,” and “negative” refer to judged feelings about the RPs. RPs = reference points; LA = loss averse, RA = risk averse, GS = gain seeking; RS = risk seeking.

#### Individual differences

Table 8 shows pairs of reasons for participants who felt positively, neutral and negatively about rejecting the gamble. Because probabilities of gamble outcomes were .5, reference-point theory predicts that risk averters should not be OGSs, and risk seekers should not be PLAs. There were some violations; 0%, 3%, and 9% of risk averters who had positive, neutral, and negative feelings about gamble rejection were OGSs. In addition, 6%, 3%, and 0% of risk seekers who felt positive, neutral, and negative about gamble rejection were PLAs. Some of these violations seem large, but when we average over affective categories, percentages were smaller (6% of risk averters and 5% of risk seekers).

**Table 8.** Reasons for Risk Preferences With Positive, Neutral, and Negative Feelings About Reference Points

	Risk averters				Risk seekers			
	PLA	PGS	OLA	OGS	PLA	PGS	OLA	OGS
Positive RPs (*N* = 482)	74%	0%	7%	0%	6%	1%	7%	4%
Neutral RPs (*N* = 426)	31%	8%	2%	3%	3%	21%	1%	30%
Negative RPs (*N* = 219)	0%	27%	0%	9%	0%	6%	0%	57%

Note: “Positive,” “Neutral,” and “Negative” refer to judged feelings about the RPs. RPs = reference points; PLA = pessimistic loss averter; PGS = pessimistic gain seeker; OLA = optimistic loss averter; OGS = optimistic gain seeker.

What were the most common reasons behind risk preferences? When decision makers felt good about rejecting the gamble, risk averters were often PLAs (74%), and risk seekers were frequently OLAs (7%). Both groups tended to be loss averse. When decision makers felt bad about rejecting the gamble, risk averters were often PLAs (20%), closely followed by PGSs (16%), and risk seekers were both OLAs (20%) and OGSs (20%). Both groups were loss averse and gain seeking. For some, hedonic sensitivities were consistent with the valence of the reference point.

#### Predicting risk preferences

Finally, we fit reference-point theory to the data. For 5% of participants, decision weights were outside the range of 0 to 1. An additional 9% of participants had judged feelings about options that were inconsistent with their choice. Finally, 16% gave choice options the same rating, so we assumed that 8% could be described by reference-point theory and that 8% could not. Thus, 22% (9% + 5% + 8%) of participants could not be described by the theory, or 78% were accurately predicted.

Prospect theory predicts that decision makers dislike fair 50/50 gambles and correctly accounted for 59% of participants. A comparison of the theories showed that reference-point theory accounted for a greater percentage of participants than prospect theory (78% vs. 59%), *t*(1198) = 14.22, *p* < .001.

## General Discussion

Theories of risk preferences usually assert that the reference point is the status quo. We define the reference point as the riskless option. The sign of one’s feelings associated with the reference point predicts one’s hedonic sensitivities. When decision makers feel good about the reference point, they foresee greater pain from a negative change than pleasure from an improvement, consistent with loss aversion. When they feel bad about the reference point, they anticipate greater pleasure from an improvement than pain from a comparable decline, consistent with gain seeking. Changes in hedonic sensitivities can thus motivate risk aversion or risk seeking. Our results mesh well with those of Voichek and Novemsky (2021), who found that decision makers with more desirable reference points tended to be risk averse, and those with less desirable reference points tended to be risk seeking.

We are hardly the first to consider nonneutral reference points. Others have examined reference points with affective charge in situations involving aspiration levels (Heath et al., 1999), minimal acceptable thresholds (March & Shapira, 1992), social comparisons (Morewedge et al., 2019), and past expectations (Koszegi & Rabin, 2006). Indeed, Kahneman and Miller (1986) treated norms as reference points. Norms are created when people recruit mental representations to explain events, something they often do when they imagine how an unusually bad outcome could have been avoided. But nonneutral reference points are unusual in theories of risky choice.

### Which other theories account for the data?

Prospect theory is the most likely contender to reference-point theory, and these theories differ in important respects. First, reference-point theory says the reference point is the riskless option. Prospect theory says the reference point is the status quo. Second, in reference-point theory, feelings about the reference point can pleasurable or painful. In prospect theory, the reference point is neutral. Third, prospect theory assumes that decision makers are described by one utility function and one decision-weighting function. Reference-point theory allows individuals to differ in their utilities and decision weights. Fourth, in its current form, reference-point theory addresses only choices between sure things and gambles, whereas prospect theory makes predictions for a much wider range of choices.

**Fig. 5.**
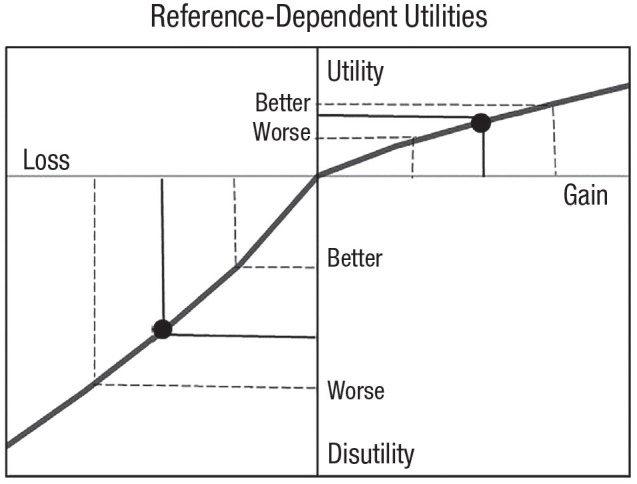
Allowing the reference point to vary in prospect theory (dots). Dashed lines show equidistant changes around each reference point. With a positive reference point, loss aversion occurs, and with a negative reference point, gain seeking emerges. From Mellers et al. (2021).

Despite these differences, there is a way for the prospect-theory utility function to account for changes in loss aversion and gain seeking due to the reference point. If the reference point can shift along the utility function, prospect theory captures both loss aversion and gain seeking, as shown in Figure 5. When the reference point has positive utility, negative changes have greater impact than comparable positive changes, consistent with loss aversion. But when the reference point has negative utility, the concave upward utility function predicts that positive changes have greater impact than negative changes, consistent with gain seeking.

**Fig. 6.**
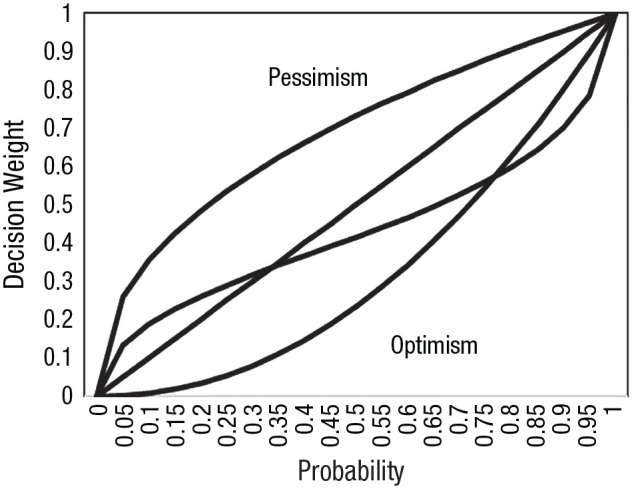
Illustration of prospect theory’s decision-weighting function and pessimistic and optimistic decision-weighting functions.

Simply relaxing the reference-point assumption in prospect theory is not enough to account for our data. Individuals also need different decision-weighting functions. Figure 6 shows the additional curves needed for prospect theory to account for the data—a concave downward curve for pessimists and a concave upward curve for optimists.

We do not want to suggest that Kahneman and Tversky (1979) thought all reference points were neutral. They wrote,

Gains and losses, of course, are defined relative to some neutral reference point. The reference point usually corresponds to the current asset position, in which cases gains and losses coincide with actual amounts that are received and paid. However, the location of the reference point, and the consequent coding of outcomes as gains or losses, can be affected by the formulation of the offered prospects, and by the expectations of the decision maker. (p. 275)

But changes of reference points were never formally developed.

Reference-point theory is similar to security-potential/aspiration (SP/A) theory (Lopes, 1984, 1987; Lopes & Oden, 1999) because it reflects optimism (potential) and pessimism (security) in the decision-weighting function. People either assess gamble outcomes from the bottom up, starting with the worst outcome (security-minded), or from the top down, starting with the best outcome (potential-minded). However, reference-point theory and SP/A theory differ, especially about reference points. In our account, the reference point is the riskless option, and in SP/A, it is the aspiration (an outcome that is at or above a subjective threshold). SP/A theory is unable to predict effects of the valence of the reference point.

Reference-point theory is also similar to salience theory (Bordalo et al., 2012). In salience theory, decision makers attend to the most salient payoffs, and the decision-weighting function assigns disproportional weight to the most salient states of the world. Payoffs in a given state of the world are salient if they differ from each other. Salience theory predicts that decision weights depend on both the rank order of the outcome and the magnitudes of the outcomes relative to others. Reference-point theory posits that people place greater weight on salient outcomes, but weights depend only on beliefs about risk. The theories also differ in assumptions about the reference point. In our account, the reference point is the riskless option, whereas in salience theory, it is zero. Like SP/A theory, salience theory cannot account for changes in risk preferences associated with the valence of the reference point.

Taking a foraging perspective, Mishra and Fiddick (2012) suggested the reference point is the decision maker’s desired state. When the disparities between decision makers’ present states and desired states are large, decision makers will take more risks. When disparities are small, they will take fewer risks. Although Mishra and Fiddick did not mention the valence of the reference point, decision makers are likely to have fewer needs when the reference point is good and more needs when the reference point is bad. In that respect, their account resembles ours.

### Correlates of risk preferences

Numerous studies have explored the demographic and personality correlates of risk preferences. Several studies have reported that women are more risk averse than men (Bryne et al., 1999; Eckel & Grossman, 2008), but not all. Flippin and Crosseto (2016) conducted a meta-analysis of gender differences and argued that effect sizes for differences between men and women are task specific. Age is also a correlate of risk preferences. Studies have shown that adults are more risk averse than young people (Banks et al., 2020; Dohmen et al., 2017). Using our method, researchers could explore the reasons for both gender and age differences across different tasks and domains.

Laboratory studies have shown that risk preferences are influenced by prior gains and losses (Thaler & Johnson, 1990; M. Weber & Zuchel, 2005), and field studies have shown that large negative events can influence risk attitudes. Abatayoa and Lynham (2020) conducted a study of fishermen on a remote Philippine island after a typhoon destroyed coral reefs and reduced fish populations. People who were hit directly by the typhoon and experienced greater losses were more risk seeking than people who were not directly hit.

Wealth is associated with risk preferences. l’Haridon and Vieider (2019) measured risk preferences in 30 countries. People in poorer countries are more risk seeking than those in richer countries. Finally, political ideology has been linked to risk preferences; conservatives are more risk averse than liberals (Choma et al., 2014; Kam, 2012). Our procedure would permit researchers to find out how liberals and conservatives differ—hedonic sensitivities, risk beliefs, or both?

Moods influence risk preferences. For example, fear encourages risk aversion, and fearful people are more pessimistic about risk (Campos-Vazquez & Cuilty, 2014; Lerner et al., 2003). Likewise, anger promotes risk seeking, and angry people are more optimistic about risk (Lerner & Keltner, 2001). Do hedonic sensitivities change with moods? Our framework allows researchers to find out.

### Predicting the risk preferences of others

When people imagine the risk preferences of others, they consider how others’ preferences differ from their own. Hsee and Weber (1997) found that risk averters predicted that others were less risk averse than they were, especially when the risks were described abstractly. Faro and Rottenstreich (2006) replicated the results and showed that risk seekers thought other people were less risk seeking than they were. Predictions of others appear to regress toward the mean of risk neutrality relative to their own risk preferences. Reference-point theory provides a way to explore the reasons for these deviations.

In closing, reference-point theory is currently limited to choices between sure things and binary gambles (or choices to accept or reject fair 50/50 gambles). The reference point is the outcome that occurs if risk is rejected. One way to generalize the theory is to assume the reference point is the less risky option. When people face choices between two risky options, the less risky option might may be the reference point, as formalized by Koszegi and Rabin (2006). Reference points are often charged with affect, and that affect is a reliable predictor of hedonic sensitivities and risk preferences. By integrating the valence of the reference point into theories of risk preference and allowing individuals to differ in their hedonic sensitivities and beliefs about risk, researchers can better understand when and why decision makers seize or sidestep risk.

## Supplemental Material

sj-docx-1-pps-10.1177_17456916231190393 – Supplemental material for Reference-Point Theory: An Account of Individual Differences in Risk Preferences Supplemental material, sj-docx-1-pps-10.1177_17456916231190393 for Reference-Point Theory: An Account of Individual Differences in Risk Preferences by Barbara A. Mellers and Siyuan Yin in Perspectives on Psychological Science

## Transparency

*Action Editor:* Tim Pleskac

*Editor:* Interim Editorial Panel

## Notes

1. Although reference-point theory predicts that risk preferences are driven by hedonic sensitivities and beliefs about risk, Equation 1 shows feelings about the gamble using outcomes, not changes from the reference point. This is not a problem; we can convert Equation 1 into an expression with hedonic sensitivities and beliefs if we subtract feelings about the sure thing from both options. That is, if F(G) – F($500) > 0, decision makers should select the gamble, and if the opposite occurs, they should select the sure thing. Now the risky option becomes:

w×|F($250)−F($500)|+(1−w)×|F($750)−F($500)|,

where *w* is the decision weight of the negative change and 1 – *w* is the weight of the positive change.

2. If values of zero represent the status quo in prospect theory, the outcomes of seven of the eight choices are in the gain domain. The exception is the choice between a sure job with an average winter temperature of –10° F and the risky job with 50/50 chances that average winter temperatures will be 30° F or –10° F. This is a mixed gamble, but it is possible that prospect theory still predicts risk aversion.

3. In this table, we refer to “PLAs” as justpessimistic loss averters, although reference point theory predicts that pessimists with equal hedonic contrasts, and calibrated loss averters (participants with one or two reasons for risk aversion) should also be risk averse. Likewise, “OGSs” refers to just optimistic gain seekers, although optimists with equal hedonic contrasts, and calibrated gain seekers (participants with one or two reasons for risk seeking) should also be gain seeking.

4. In Mellers et al. (2021), reference points for fair 50/50 gambles were baseline feelings about how people felt before seeing the gamble. Here, we have a more specific reference point as defined by the feeling associated with rejecting the gamble.
