# Supplementary material for: Reference-Point Theory: An Account of Individual Differences in Risk Preferences
Source: Perspect Psychol Sci. 2023 Sep 14;20(1):99–114. doi: 10.1177/17456916231190393 (PMC11720267; doi:10.1177/17456916231190393)
Supplement: sj-docx-1-pps-10.1177_17456916231190393 – Supplemental material for Reference-Point Theory: An Account of Individual Differences in Risk Preferences [file sj-docx-1-pps-10.1177_17456916231190393.docx]

**Appendix: Table of Contents**

1. Demonstration that Hedonic Contrasts and Beliefs about Outcomes are Independent
2. Predictions of Reference-Point Theory for Risk Prefernces
3. Hedonic Sensitivities and Risk Preferences for each Life Dimension in Study 1
4. Assumptions of Reference-Point Theory in the Disease Problem in Study 2

**1. Demonstration that Hedonic Contrasts and Beliefs about Outcomes are Independent**

In this section, we show that four hypothetical judged feelings can produce in nine sets of reasons for risk prefences. Judged feelings are F(G), F(RP, F(b) and F(w) for the gamble, the reference point (sure thing), the best outcome and the worst outcome, respectively. Suppose decision makers rate their feelings about options and outcomes on a rating scale from 1 (Very Painful) to 7 (Very Pleasurable). Ratings appear in Columns 1 through 4 of Table 1A. Columns 5 and 6 are differences between feelings about gamble outcomes and the reference point. Column 7 indicates whether the decision maker is loss averse or gain seeking (i.e., is Column 5 is greater or less than Column 6). Columns 8 and 9 are the numerator and denominator of the weight of the worst outcome in the gamble, respectively. Column 10 shows the decision weight. In this example, we hold constant feelings about better and worse outcomes (6 and 2, respectively), so the average is always 4. We vary only F(RP) and F(G). Table 1A demonstrates the pairs of reasons and shows that hedonic sensitivities and beliefs about risk are independent.

**Table 1A.**

**2. Predictions of Reference-Point Theory for Each Risk Profile**

Here we show how reference-point theory constrains predicted risk preferences. Define Av = [F(b) + F(w)]/2, the average of feelings about gamble outcomes. Loss aversion means that F(RP) – F(w) > F(b) – F(RP). This means 2*F(RP) > F(l) + F(g), or F(RP) > [F(l) + F(g)]/2. Loss aversion implies that F(RP) > Av. Gain seeking implies that F(RP) < Av, and equal hedonic contrasts implies that F(RP) = Av.

Suppose gamble probabilities are .5. Pessimism about risk means [F(G) – F(b)]/[F(w) – F(b)] > .5. This can be written as F(G) – F(b) < .5*[F(w) – F(b)] or F(G) < [F(w) + F(b)]/2 = Av. Pessimism about risk implies that F(G) < Av. Optimism about risk implies that F(G) > Av and calibration means F(G) = Av.

Reference-point theory predicts that, if F(RP) > F(G), the individual is risk averse. If F(G) > F(RP), the individual is risk seeking, and if F(G) = F(RP), the individual is indifferent. Now we’ll explore implied risk preferences for each risk profile.

Loss Averse and Pessimistic: Loss aversion implies F(RP) > Av and pessimism implies F(G) < Av or Av > F(G). By transitivity, F(RP) > F(G). Pessimistic loss-averters are risk averse.

Loss Averse and Calibrated: Loss aversion implies F(RP)> Av and calibration means F(G) = Av. If F(RP) > Av and Av = F(G), F(RP) > F(G). Calibrated loss averters should be risk averse.

Loss Averse and Optimistic: Loss aversion implies F(RP) > Av and optimism implies F(G) > Av. There are no constraints on the rank order of F(G) and F(RP). Optimistic loss averters can be either risk averse or risk seeking.

Equal Contrasts and Pessimistic: Equal Contrasts mean F(RP) = Av. Pessimism means F(G) < Av or Av > F(G). By transitivity, F(RP) > F(G), so pessimistic decision makers with equal contrasts should be risk averse.

Equal Contrasts and Calibrated: Equal contrasts imply F(RP) = Av. Calibrated weights imply F(G) = Av. For these decision makers, F(RP) = F(G). Decision makers are indifferent.

Equal Contrasts and Optimistic: Equal contrasts imply F(RP) = Av. Optimism implies that F(G) > Av. This implies F(G) > F(RP), so these decision makers should be risk seeking.

Gain Seeking and Pessimistic: Gain seeking implies Av > F(RP) and pessimism means Av > F(G). There are no constraints on the rank order of F(G) and F(RP), pessimistic gain-seekers can be either risk averse or risk seeking.

Gain-Seeking and Calibrated: Gain seeking implies Av > F(RP) and calibration means F(G) = Av. If Av > F(RP) and Av = F(G), then F(G) > F(RP), so calibrated gain-seekers should be risk seeking.

Gain Seeking and Optimistic: Gain seeking implies Av > F(RP) and optimism implies F(G) > Av. If F(G) > Av and Av > F(RP), then F(G) > F(RP). Optimistic gain-seekers should be risk seeking. Assumptions and implications are summarized in Table 2A.

**Table 2A.**

Note: Rows are pairs of reasons. The first two columns show the implications of pairs of reasons based on HS (Hedonic Sensitivities) and BEL (Beliefs about Risk). The next two columns show what HS and BEL imply and the predicted risk preference(s) for that pair of reasons.

**3. Hedonic Sensitivities and Risk Preferences for each Life Dimensions in Study 1**

Here, we show hedonic contrasts, beliefs and risk preferences for each life dimension and each reference point condition separately.

**Commute**

**“Positive” RPs** **LA RA GS RS**

Better (72) 92% 90% 6% 10%

Worse (28) 86% 82% 14% 18%

**Average (100) 89% 88% 10% 12%**

**“Neutral” RPs LA RA GS RS**

Better (18) 17% 83% 33% 17%

Worse (14) 43% 79% 29% 21%

**Average (32) 29% 81% 30% 19%**

**“Negative” RPs LA RA GS RS**

Better (7) 14% 57% 86% 43%

Worse (55) 7% 51% 82% 49%

**Average (62) 11% 52% 84% 48%**

Differences between LA, RA, GS and RS were statistically significant when responses toward the reference point were positive vs negative. Tests were t(160)=4.86, 9.65,9.17 and -4.85, respectively.)

**Safety**

**“Positive” RPs** **LA RA GS RS**

Better (90) 51% 52% 14% 48%

Worse (78) 72% 73% 28% 27%

**Average (168) 61% 62% 21% 38%**

**“Neutral” RPs LA RA GS RS**

Better (2) 0% 0% 100% 100%

Worse (12) 0% 17% 8% 83%

**Average (14) 0% 14% 21% 86%**

**“Negative” RPs LA RA GS RS**

Better (0)

Worse (5) 20% 100% 40% 0%

**Average (5) 20% 100% 40% 0%**

Differences between LA, RA, GS and RS were statistically significant when responses toward the reference point were positive vs negative, with the exception of GS. Tests were t(170) = 2.13, 1.84, 1.01 and -2,13, respectively.)

**Salary**

**“Positive” RPs** **LA RA GS RS**

Better (87) 98% 95% 0% 5%

Worse (69) 100% 91% 0% 9%

**Average (156) 99% 94% 0% 6%**

**“Neutral” RPs LA RA GS RS**

Better (12) 73% 83% 25% 17%

Worse (15) 42% 80% 7% 20%

**Average (27) 59% 81% 15% 19%**

**“Negative” RPs LA RA GS RS**

Better (3) 33% 67% 67% 33%

Worse (16) 19% 63% 69% 37%

**Average (19) 21% 63% 68% 37%**

Differences between LA, RA, GS and RS were statistically significant when responses toward the reference point were positive vs negative. Tests were t(173) = 3.13, 6.55, 5.99 and -3,13, respectively.)

**Temperature**

**“Positive” RPs** **LA RA GS RS**

Better (65) 82% 66% 11% 34%

Worse (27) 81% 70% 11% 30%

**Average (92) 82% 67% 11% 33%**

**“Neutral” RPs LA RA GS RS**

Better (18) 50% 61% 39% 39%

Worse (21) 62% 67% 14% 33%

**Average (39) 56% 64% 26% 36%**

**“Negative” RPs LA RA GS RS**

Better (14) 0% 29% 100% 71%

Worse (47) 19% 60% 68% 40%

**Average (61) 15% 52% 75% 48%**

Differences between LA, RA, GS and RS were statistically significant when responses toward the reference point were positive vs negative. Tests were t(150) = 1.85, 8.12, 7.85 and -1.85, respectively.)

**4. Assumptions of Reference-Point Theory in the Disease Problem in Study 2**

Reference-point theory makes no predictions about reasons for risk preferences because, with the probability of the worst outcome being .67 rather than .5, inequalities are less constrained. To illustrate, let’s examine the lives saved frame where RP = 200 lives saved. Loss aversion implies that F(200 LS) – F(0 LS) > F(400 LS) - F(200 LS) or F(200 LS) > [F(0 LS) + F(400 LS)]/2. Define Av = [F(0 LS) + F(400 LS)]/2. Then loss aversion means F(RP) > Av, gain seeking means F(RP) < Av and equal hedonic contrasts mean F(RP) = Av.

Pessimism implies that [F(G) – F(600 LS)]/[F(0 LS) – F(600 LS)] > .67. We can rearrange the equation as follows, F(G) < .67*[F(0 LS) – F(600 LS)] + F(600 LS) or F(G) < [2*F(0 LS) + F(600 LS)]/3. Define Av^* =^ [2*F(0 LS) + F(600 LS)]/3, an unequal weighting of better and worse outcomes, not the simple average. Since Av differs from Av*, there are no constraints for risk preferences in each risk profile.
